# A deeper dip? Hot‐water immersion and nocturnal blood pressure regulation

**DOI:** 10.1113/EP093711

**Published:** 2026-02-27

**Authors:** Brendon H. Roxburgh, Kate N. Thomas

**Affiliations:** ^1^ School of Physical Education, Sport and Exercise Sciences University of Otago Dunedin New Zealand; ^2^ HeartOtago, University of Otago Dunedin New Zealand; ^3^ Department of Surgery and Critical Care University of Otago Dunedin New Zealand

**Keywords:** ambulatory blood pressure measurement, blood pressure, hot‐water immersion, nocturnal dipping

1

Hot‐water immersion (HWI) is increasingly promoted as a simple, non‐pharmacological strategy to improve cardiovascular health (Roxburgh et al., 2026). Acute laboratory studies consistently demonstrate substantial reductions in diastolic blood pressure during and immediately after heat exposure (Price et al., 2025); effects on systolic blood pressure appear more variable. However, whether any acute responses translate into meaningful changes in daily blood pressure regulation remains unclear. In this issue of *Experimental Physiology*, Leaney et al. (2026) provide a timely and methodologically robust examination of this question using 24 h ambulatory blood pressure monitoring in healthy adults.

The principal finding is straightforward, in that a single morning session of HWI produces marked reductions in diastolic and mean arterial blood pressure during immersion and in the immediate post‐heating period. However, it does not reduce 24 h, daytime or nighttime ambulatory systolic or diastolic blood pressure. Although HWI increased the nocturnal diastolic blood pressure dip, systolic blood pressure (the most prognostically important component of arterial pressure) was unaffected.

Much of the enthusiasm surrounding HWI stems from reductions in blood pressure measured in controlled laboratory conditions, often immediately after heat exposure. The present findings reinforce that such reductions, although physiologically real, do not necessarily persist into daily life in young, normotensive adults. This distinction matters. Ambulatory systolic blood pressure is a stronger predictor of cardiovascular events than clinical or resting measures, and its resistance to change here suggests that a single bout of HWI is unlikely to confer acute cardiovascular benefit in healthy individuals.

A major strength of this study is the use of 24 h ambulatory blood pressure monitoring and should serve as an exemplar for methodological rigour and transparent reporting of ambulatory blood pressure assessment in heat therapy research. A particular hat tip is warranted for the participants, who consented to frequent measurements every 20 min during the day (and every 30 min overnight), substantially enhancing the robustness of the ambulatory data and confidence in these conclusions.

The observed greater magnitude of nocturnal diastolic blood pressure dipping following HWI in young, healthy adults is statistically robust but clinically ambiguous. Blunted nocturnal dipping is associated with increased cardiovascular risk, particularly in hypertensive and clinical populations (Salles et al., 2016). In patients with autonomic failure, who commonly exhibit supine hypertension and an absent nocturnal dipping pattern, Okamoto et al. (2021) demonstrated that local passive heating during sleep (via a heated bed mat) produces large reductions in nocturnal systolic blood pressure and improved morning orthostatic tolerance; an outcome that, if repeated, would probably translate to reduced cardiovascular risk.

In contrast, in young, healthy adults with intact autonomic control and blood pressure in a healthy range, modestly augmented nocturnal diastolic dipping (3.7%) in the absence of concomitant reductions in systolic blood pressure are more difficult to interpret clinically. The temptation to over‐interpret isolated changes in diastolic patterns should be resisted, particularly when systolic pressure, the dominant determinant of risk, remains unchanged.

Notably, many participants exhibited an ‘extreme’ diastolic dipping pattern (>20% reduction below daytime levels) following HWI. Some cohort studies report a U‐shaped association between the magnitude of nocturnal blood pressure dipping and future subclinical cardiovascular risk, whereby both blunted and excessive dipping are linked with increased prevalence of markers such as coronary artery calcium in young adults (Viera et al., 2012). However, evidence specifically isolating excessive nocturnal diastolic dipping as a prognostic factor, especially in healthy, young populations, remains limited and requires further investigation. Repeated HWI exposures, alternative timing (e.g., evening heating) and targeted studies in populations with elevated nighttime blood pressure or blunted nocturnal dipping might yet reveal clinically meaningful effects.

Certain methodological choices warrant discussion. Blood pressure during HWI was initially assessed 15 min into immersion. Experimental evidence suggests that in young, healthy individuals, systolic blood pressure often falls rapidly upon immersion, reaching a nadir within the first few minutes before partly recovering despite continued thermal stress (Menzies et al., 2025). In our anecdotal observations, the blood pressure of older, frailer or clinically vulnerable individuals tends to decline progressively throughout heating, reflecting impaired compensatory mechanisms (Figure 1). Delaying blood pressure measurement therefore risks obscuring these distinct trajectories. Time‐averaged or late‐phase measurements might underestimate the magnitude of hypotension induced. Although a brief exaggerated drop in this population is likely to be trivial, particularly in light of the work by Leaney et al. (2026), from a mechanistic perspective, the early phase of heat exposure (when rapid vasodilatation challenges central blood pressure regulation) might be an informative window for understanding cardiovascular control during thermal stress.

**FIGURE 1:**
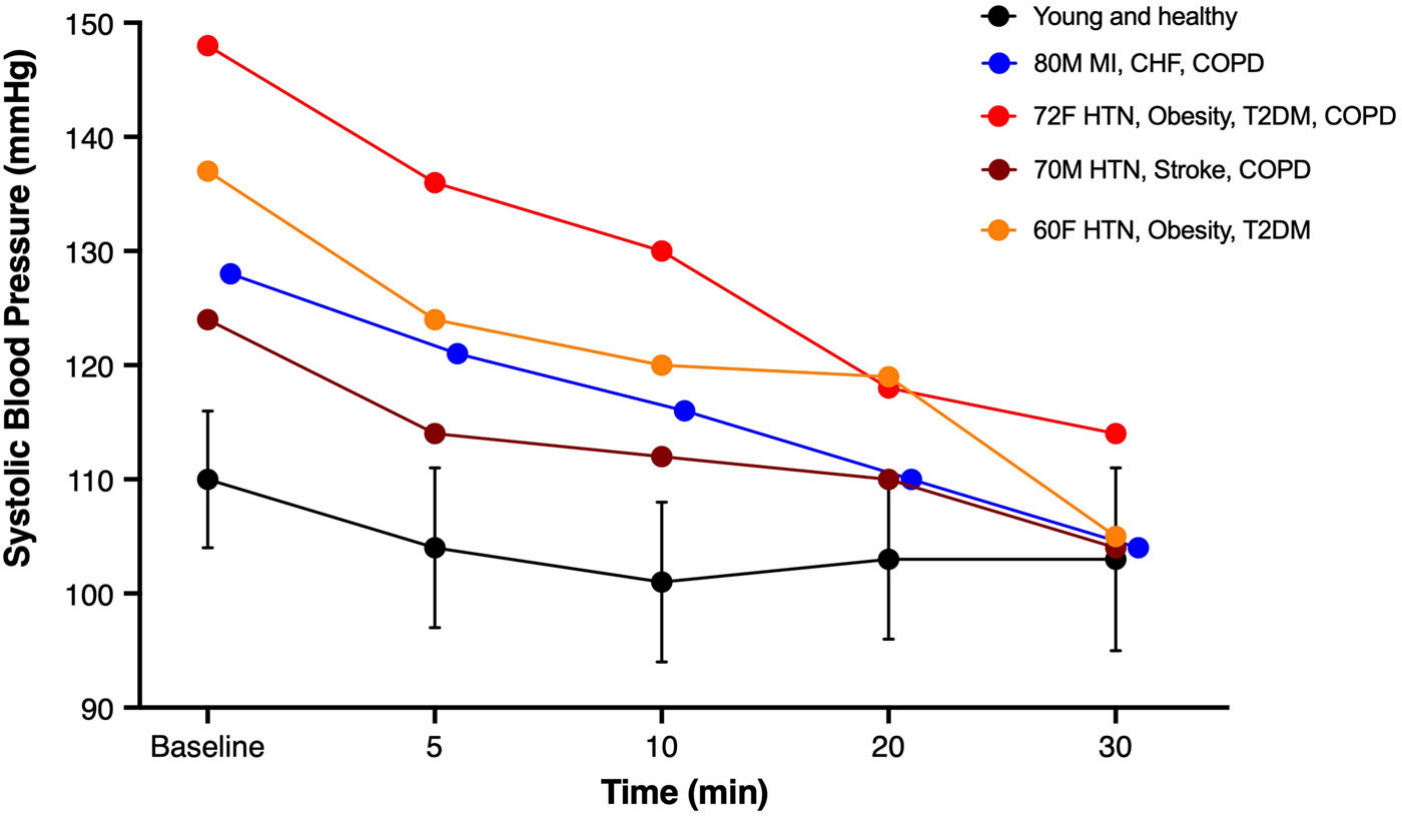
Systolic blood pressure during acute chest‐deep hot‐water immersion (40°C) in young, healthy adults (*n* = 12; age 31 ± 15 years; unpublished data) and in individual older adults with multiple comorbidities reported in a previous study (Roxburgh et al., 2023). Older adults are presented as individual cases. Data are mean ± SD or absolute values, as appropriate. Abbreviations: CHF, chronic heart failure; COPD, chronic obstructive pulmonary disease; F, female; HTN, hypertension; M, male; MI, previous myocardial infarction; T2DM, type 2 diabetes mellitus.

As the authors have acknowledged, the absence of a thermoneutral water immersion control is another important consideration. Although the air control condition isolates the effect of heat exposure relative to rest, it does not account for the haemodynamic consequences of water immersion itself that have effects on ambulatory blood pressure (Roxburgh et al., 2025). Immersion per se increases central blood volume and cardiac preload via hydrostatic pressure, effects that might partly offset heat‐induced reductions in systemic vascular resistance (Roxburgh et al., 2026). Without a thermoneutral immersion comparator, it is difficult to distinguish the independent contributions of heat and immersion to the observed blood pressure responses. However, we appreciate that the chosen design represents a pragmatic, realistic sedentary comparison.

Leaney et al. (2026) provide a valuable contribution to the field, i.e., that reductions in arterial blood pressure during HWI do not necessarily translate into lower ambulatory blood pressure over the subsequent 24 h in young, healthy adults. Although the enhancement of nocturnal diastolic dipping is intriguing, its clinical relevance in this population remains uncertain. Future research should continue to focus on the blood pressure response following HWI, how any phenotypic responses differ, and ambulatory outcomes that reflect cardiovascular risk meaningfully.

## AUTHOR CONTRIBUTIONS

Brendon H. Roxburgh and Kate N. Thomas contributed to the conception of the Viewpoint. Each author participated in designing, writing and revising the Viewpoint, approved the final version and agrees to be accountable for all aspects of the work in ensuring that questions related to the accuracy or integrity of any part of the work are appropriately investigated and resolved. Both persons designated as authors qualify for authorship, and all those who qualify for authorship are listed.

## CONFLICT OF INTEREST

None declared.

## FUNDING INFORMATION

None.
